# Triglyceride-glucose index is associated with hypertension incidence up to 13 years of follow-up in mexican adults

**DOI:** 10.1186/s12944-023-01925-w

**Published:** 2023-09-27

**Authors:** Anna D. Argoty-Pantoja, Rafael Velázquez-Cruz, Joacim Meneses-León, Jorge Salmerón, Berenice Rivera-Paredez

**Affiliations:** 1 Programa de Maestría y Doctorado en Ciencias Médicas, Odontológicas y de la Salud. Facultad de Medicina, Universidad Nacional Autónoma de México (UNAM), Ciudad de México, 04510 México; 2 Laboratorio de Genómica del Metabolismo Óseo, Instituto Nacional de Medicina Genómica (INMEGEN), Ciudad de México, 14610 México; 3 Centro de Investigación en Políticas, Población y Salud (CIPPS), Universidad Nacional Autónoma de México (UNAM), Ciudad de México, 04510 México

**Keywords:** Triglyceride-glucose index, Blood pressure, Hypertension, Incidence, Mexican population

## Abstract

The triglyceride-glucose index (TyG index) is an indicator of insulin resistance that has been studied recently. The relationship between insulin resistance and the risk of hypertension has been documented previously. However, there is limited knowledge regarding the association of the TyG index with hypertension incidence. This study aimed to evaluate the association of the TyG index with changes in blood pressure (BP) and hypertension incidence in Mexican adults. This analysis was performed using the Health Workers Cohort Study data. The TyG index was estimated as Ln [fasting triglycerides (mg/dL) × fasting glucose (mg/dL)/2] and divided into categories defined by tertiles. The analysis was conducted using fixed-effects linear regression models (n = 1,545) and Cox proportional hazards regression models (n = 1,113), adjusting for potential confounding variables. The incidence rates (95% CI) for the low, medium, and high categories of the TyG index were 22.1 (17.8, 27.5), 35.8 (30.1, 42.7), and 49.4 (42.1, 57.9), respectively. An increase in the levels of systolic blood pressure (SBP) and diastolic blood pressure (DBP) was observed when changing from a low to a medium (DBP: β 2.55 mmHg, 95% CI 0.81, 4.29) and from a low to a high category of the TyG index (SBP: β 3.10 mmHg, 95% CI 1.16, 5.04; DBP: β 4.91 mmHg, 95% CI 2.88, 6.94). Furthermore, participants within the top category of the TyG index had a 56% higher risk of hypertension than those in the bottom category (HR = 1.56; 95% CI 1.18, 2.08). These results support the hypothesis that the TyG index is associated with high blood pressure in Mexican adults.

**Supplementary Information:**

The online version contains supplementary material available at 10.1186/s12944-023-01925-w.

## Introduction

Hypertension affects over 30% of adults worldwide, representing an important risk factor for other cardiovascular outcomes and premature death [1]. The hypertension prevalence in Mexican adults increased by approximately 7% from 2012 to 2018 (27.2% vs. 34.1%, respectively) [2]. Hypertension is caused by different factors, including genetics, lifestyle, and metabolic factors, including obesity, dyslipidemia, diabetes, and insulin resistance [3, 4]. A recent meta-analysis of 11 observational studies with 55,059 subjects demonstrated that insulin resistance was associated with hypertension development [5]. Insulin resistance is defined as the “inability of insulin to increase cellular glucose uptake and utilization, leading to compensatory hyperinsulinemia” [5]. Its significance is crucial in hypertension pathophysiology through enhanced tissue angiotensin II and aldosterone functions, increased sympathetic nervous system activity, and contributes to oxidative stress [6, 7]. Because insulin and C-peptide assays are difficult to perform, a basic indicator that identifies insulin resistance could benefit clinical hypertension management [8]. The triglyceride-glucose index (TyG index) is defined as the product of fasting triglycerides and glucose levels, and it is a reliable substitute indicator of insulin resistance [9]. It has shown a strong correlation with the total glucose metabolism rates in the hyperinsulinemic–euglycemic clamp test [9–11]. A meta-analysis of 8 observational studies indicated the association between the TyG index and hypertension; most studies were conducted in China, six were cross-sectional and two were longitudinal studies [12]. None of the previous studies included diet as a confounder, and only one study (cross-sectional) included lipid-lowering medications in the analysis [12–14]. Given the limitations of previous studies, such as cross-sectional evaluation and residual confounding, it is necessary to conduct prospective cohort studies to confirm these results and deepen this phenomenon. In addition, whether the TyG index is longitudinally associated with phenotypes related to hypertension has not been evaluated in the Mexican population. Therefore, the aim of this study was to evaluate the association between the TyG index and changes in blood pressure (BP) and hypertension incidence in Mexican adults.

### Methods

#### Study population

A longitudinal analysis was conducted with data derived from the Health Workers Cohort Study (HWCS). The HWCS is a dynamic cohort conducted in Central Mexico to evaluate the association between lifestyle and genetic factors with several health outcomes. Subjects of this cohort are employees and their relatives from the Mexican Social Security Institute (IMSS, by its acronyms in Spanish) [15]. For the present study, data from three follow-up waves were used: 2004–2006, 2010–2012, and 2016–2019. A total of 1,593 participants aged ≥ 20 years with at least two measurements were included. Participants with missing BP values (n = 27) and missing TyG index values (n = 21) were excluded. The final sample for evaluating BP changes was 1,545 subjects, and for evaluating hypertension incidence, it was 1,113 subjects (432 participants with hypertension at baseline were excluded) (Figure S1).

### Blood pressure and hypertension

Trained nurses used an automatic monitor (OMROM HEM-907) to measure systolic blood pressure (SBP) and diastolic blood pressure (DBP) following standard procedures from the World Health Organization (WHO) [16, 17]. Incident hypertension was defined by meeting one of the following criteria at the follow-up assessment (2012–2012 or 2016–2019) in those participants who were free of hypertension at baseline: 1) self-reported medical diagnosis (participants reported the year of medical diagnosis), 2) taking antihypertensive medication (participants reported the medications they were taking on the date of the survey), or 3) SBP/DBP ≥ 140/> 90 mmHg (blood pressure levels measured on the date of the survey). The time/period used to define a new case was determined as half of the reported year of medical diagnosis or the date at the survey diagnosis.”

### Triglyceride-glucose (TyG) index

The TyG index was estimated as Ln [fasting triglycerides (mg/dL) × fasting glucose (mg/dL)/2] [10]. The TyG index was divided into low, medium, and high categories according to cutoffs derived from tertiles. A blood sample after a fasting period of ≥ 8 h was taken from each participant, and chemistry tests were performed [15]. Glucose levels were measured with the oxidized glucose method, and triglycerides were obtained through a colorimetric method after enzymatic hydrolysis with the lipase technique [18]. The Selectra XL instrument (Randox) was used to carry out the biomedical assays following the International Federation of Clinical Chemistry and Laboratory Medicine [15, 18].

### Covariate assessment

Demographic information (age and sex), medication intake (antihypertensive, hypoglycemic, and lipid-lowering), family history of hypertension, and lifestyle information (dietary intake, physical activity, smoking and alcohol intake) were collected from a self-administered questionnaire at each study wave [15]. Smoking status was categorized as never, former, and current smokers. Dietary intake information was evaluated using a 116-item semiquantitative food frequency questionnaire (FFQ) validated in the Mexican population [15, 19]. Information on alcohol intake was obtained using food composition tables from the National Institute of Public Health [15]. The Dietary Approaches to Stop Hypertension (DASH) score was estimated through eight components, including consumption of fruits, vegetables, nuts and legumes, dairy products, whole grains, sodium, sweetened beverages, and red and processed meats, using a method previously reported; the score was presented on a continuous scale from 8 to 40 [20]. For the evaluation of physical activity (PA), a validated questionnaire in Spanish was used [21]. PA was categorized into two categories: inactive (< 30 min/day) or active (≥ 30 min/day) according to the World Health Organization’s definition [22]. A previously calibrated electronic scale (model BC-533, Tanita brand) was used to measure body weight, and a conventional stadiometer (brand SECA 206) was used to obtain height [15]. Body mass index (BMI) was estimated as weight (kg) divided by height (m^2^), and a BMI ≥ 30 kg/m^2^ was defined as obesity [23]. Type 2 diabetes (T2D) was defined as a self-report of physician-diagnosed T2D, antidiabetic drug intake, or fasting glucose ≥ 126 mg/dL during the examination [24, 25]. Dyslipidemia was established as having a self-reported diagnosis or lipid-lowering drug intake.

### Statistical analyses

Baseline characteristics were summarized by stratifying by sex using means and standard deviations (SD), medians, and 25th percentile (P25)-75th percentile (P75) for continuous variables and percentages for nominal and ordinal variables. The skewness-kurtosis test was used to evaluate normality. Statistical differences between categories of the TyG index were tested through the two-sample proportion test and the nonparametric Dunn’s test for categorical and numerical variables, respectively. For SBP, DBP, and the DASH diet, one-way ANOVA was performed with Bonferroni’s post hoc comparison tests. Two independent analyses were conducted: fixed-effects linear regression and Cox proportional hazards regression models.

### Fixed-effects linear regression to evaluate the association between changes in the TyG index and changes in BP levels

The change in SBP and DBP over time was estimated using unadjusted fixed-effects regression models and rescaling the time variable to 5 years. Fixed-effects linear regression models were used to assess the longitudinal association between changes in the TyG index and changes in SBP and DBP. The models were fitted by covariate changes across time, such as BMI, alcohol intake, physical activity, DASH diet, and smoking. In addition, models with lipid-lowering and hypoglycemic drugs and hypertension status were fitted.

Three sensitivity analyses were conducted: (1) excluding subjects with T2D at baseline (n = 156), (2) excluding subjects with T2D or obesity at baseline (n = 421), and (3) excluding subjects with T2D, obesity, or dyslipidemia at baseline (n = 704). The exclusions were not independent.

### Cox proportional hazards regression to evaluate the association between the TyG index and hypertension incidence rate

To assess the relationship between the TyG index and hypertension incidence, the multivariable Cox proportional hazards regression model was used. The person-years were estimated from the date of returning the baseline questionnaire to the date of self-reported medical diagnosis or follow-up visit in those with a survey diagnosis, and subjects without hypertension were censored on their final follow-up visit. The hazard ratio (HR) and 95% confidence interval (95% CI) were calculated with the low category of the TyG index as the reference group. The models were fitted by age, sex, BMI, alcohol intake, physical activity, DASH diet, smoking, and family history of hypertension.

Three sensitivity analyses were conducted: (1) excluding subjects with T2D at baseline (n = 69), (2) excluding subjects with T2D or obesity at baseline (n = 222), and (3) excluding subjects with T2D, obesity, or dyslipidemia at baseline (n = 405). The exclusions were not independent.

Statistical significance was determined by conducting two-tailed tests for all *P* values, and a threshold of *P* < 0.05 was used to indicate statistical significance. All analyses were conducted using Stata 14.1.

## Results

### Association between changes in the TyG index and changes in BP levels

This analysis included 1,545 participants with at least two measurements. A total of 768 participants had two measurements over time (mean time between measurements was 7.5 years, SD 2.5), and 777 participants had three measurements over time (mean time between the first and third measurements was 12.6 years, SD 0.9). Of the 1,545 participants, 75.7% were females. At baseline, the median age was 47 years (P25-P75: 38–57). The means of SBP and DBP were 116 mmHg (SD: 12.4) and 71 mmHg (SD: 9.7), respectively, and 27.9% of participants had hypertension. The median triglyceride level was 138 mg/dL (P25-P75: 100–196), the median glucose level was 91 mg/dL (P25-P75: 84–100), and the TyG index was 8.8 (P25-P75: 8.4–9.2) (Table 1). The cutoffs of the TyG index derived from tertiles were < 8.537, 8.538– 9.025, and > 9.025 for the low, medium, and high categories, respectively. The median BMI was 26.3 kg/m^2^ (P25-P75: 23.6–29.2), and 41.7% and 21.2% of participants were overweight and obese, respectively.

**Table 1:** Characteristics of participants in the Health Workers Cohort Study at baseline (n = 1,545)

Population characteristics	Total	Females	Males
	n = 1,545	n = 1,169 (75.7)	n = 376 (24.3)
Age, years *	47 (38–57)	48 (39–58)	45 (36–55)
Systolic blood pressure ^†,^ mmHg	116 (12.4)	114 (12.2)	120 (12.4)
Diastolic blood pressure ^†^, mmHg	71 (9.7)	71 (9.7)	73 (9.5)
Hypertension, n (%)	432 (27.9)	324 (27.7)	108 (28.7)
Triglycerides *, mg/dL	138 (100–196)	132 (96–179)	177 (115–253)
Fasting plasma glucose *, mg/dL	91 (84–100)	90 (83–98)	94 (88–103)
TyG index *	8.8 (8.4–9.2)	8.7 (8.3–9.1)	9.0 (8.6–9.5)
Body mass index *, kg/m^2^	26.3 (23.6–29.2)	26.1 (23.5–29.3)	26.7 (24.5–29.1)
Overweight, n (%)	644 (41.7)	456 (30.1)	188 (50.0)
Obesity, n (%)	327 (21.2)	253 (21.7)	74 (19.7)
Diabetes, n (%)	156 (10.1)	117 (10.0)	39 (10.4)
Dyslipidemia, n (%)	447 (28.9)	313 (26.8)	134 (35.6)
Smoking, n (%)			
Never	914 (59.2)	767 (65.6)	147 (39.1)
Former	402 (26.0)	261 (22.3)	141 (37.5)
Current	229 (14.8)	141 (12.1)	88 (23.4)
DASH diet ^†^	23.8 (4.6)	24.4 (4.9)	21.9 (4.3)
Alcohol intake *, g/day	0.8 (0.2–3.8)	0.79 (0.04–2.1)	3.9 (0.8–11.2)
Physical activity *, min/day	12.9 (3.2–36.5)	12.9 (2.7–33.2)	16.1 (4.6–46.2)
Physical activity ≥ 30 min/day, n (%)	531 (34.4)	382 (32.8)	149 (39.7)
Lipid-lowering drugs, n (%)	111 (7.2)	82 (7.0)	29 (7.7)
Hypoglycemic drugs, n (%)	75 (4.9)	54 (4.6)	21 (5.6)
Family history of hypertension			
No	354 (22.9)	245 (21.0)	109 (28.9)
Yes	1159 (75.2)	904 (77.5)	255 (67.8)
Unknown	29 (1.9)	17 (1.5)	12 (3.2)

*Median (p25-P75) †Mean (SD). TyG index: Triglyceride-glucose index. DASH diet: The Dietary Approaches to Stop Hypertension

Regarding the changes in continuous variables over a period of five years in the cohort, an increase in SBP (β = 2.88 mmHg), DBP (β = 2.34 mmHg), fasting plasma glucose (β = 5.57 mg/dL), TyG index (β = 0.07), and BMI (β = 0.49 kg/m^2^) was observed. On the other hand, decreases in the DASH diet score (β = -0.09), alcohol intake (β = -0.63 g/day), and physical activity (β = -1.93 min/day) were found (Table 2).

**Table 2:** Changes in continuous variables for every 5 years in the Health Workers Cohort Study (n = 1,545)

Population characteristics	Baseline	Change (95% CI)	*P* value
Systolic blood pressure ^†^, mmHg	116 (12.4)	2.88 (2.46, 3.29)	< 0.001
Diastolic blood pressure ^†^, mmHg	71 (9.7)	2.34 (1.91, 2.78)	< 0.001
Triglycerides *,mg/dL	138 (100–196)	1.67 (-1.23, 4.57)	0.259
Fasting plasma glucose *, mg/dL	91 (84–100)	5.57 (4.85, 6.29)	< 0.001
TyG index *	8.8 (8.4–9.2)	0.07 (0.05, 0.08)	< 0.001
Body mass index *, kg/m^2^	26.3 (23.6–29.2)	0.49 (0.41–0.58)	< 0.001
DASH diet ^†^	23.8 (4.6)	-0.09 (-0.20, 0.02)	0.103
Alcohol intake *, g/day	0.8 (0.2–3.8)	-0.63 (-1.13, -0.13)	0.014
Physical activity *, min/day	12.9 (3.2–36.5)	-1.93 (-2.73, -1.13)	< 0.001

*Median (p25-P75) † Mean (SD). TyG index: Triglyceride-glucose index. DASH diet: The Dietary Approaches to Stop Hypertension

Fixed-effects linear regression indicated that the TyG index was positively associated with SBP and DBP. The adjusted model showed that a change from a low to a high category of the TyG index increased SBP by 3.10 mmHg (95% CI 1.16, 5.04), and a change from a medium to a high category of the TyG index increased SBP by 2.26 mmHg (95% CI 0.80, 3.72) (Table 3). Additionally, this positive association was observed with DBP when changing from a low to a medium category (β = 2.55, 95% CI 0.81, 4.29), from a low to a high category (β = 4.91, 95% CI 2.88, 6.94), and from a medium to a high category of TyG index (β = 2.36, 95% CI 0.83, 3.89) (Table 3). The coefficients were maintained when adjusting for lipid-lowering and hypoglycemic drugs (Table 3). In addition, a similar association when adjusting for hypertension status was observed (data not shown).

**Table 3:** Results of the fixed effect models for the SBP and DBP change according to changes in the TyG index (n = 1,545)

	Triglyceride-glucose index*		
Systolic blood pressure	Change low to medium category β (95%CI)	Change low to high category β (95%CI)	Change medium to high category β (95%CI)
Model 1	0.84 (-0.81, 2.49)	3.10 (1.16, 5.04)	2.26 (0.80, 3.72)
Model 2	0.85 (-0.80, 2.50)	3.14 (1.20, 5.07)	2.28 (0.83, 3.73)
Model 3	0.74 (-0.91, 2.39)	2.93 (0.99, 4.86)	2.19 (0.74, 3.64)
**Diastolic blood pressure**			
Model 1	2.55 (0.81, 4.29)	4.91 (2.88, 6.94)	2.36 (0.83, 3.89)
Model 2	2.55 (0.82, 4.29)	4.93 (2.89, 6.96)	2.37 (0.85, 3.91)
Model 3	2.50 (0.77, 4.24)	4.84 (2.81, 6.88)	2.33 (0.80, 3.87)

*Categories defined by tertiles

Model 1: Model adjusted for body mass index (kg/m2), alcohol intake (g/day), physical activity (inactive/active), DASH diet, smoking (never, former, current)

Model 2: Model 1 + Lipid-lowering drugs

Model 3: Model 2 + Hypoglycemic drugs

β: regression coefficient, CI: confidence interval

The sensitivity analysis showed that the coefficients remained when excluding 156 subjects with T2D at baseline (SBP: low to a high (β = 3.75, 95% CI 1.81, 5–69) and medium to a high category (β = 3.39, 95% CI 1.91, 4.86); DBP: low to a medium (β = 2.55, 95% CI 0.66, 4.43), low to a high (β = 4.86, 95% CI 2.66, 7.05), and medium to a high category (β = 2.31, 95% CI 0.64, 3.98)). Additionally, when 421 participants with T2D or obesity at baseline were excluded (SBP: low to high (β = 2.81, 95% CI 0.77, 4.85) and medium to high (β = 3.18, 95% CI 1.61, 4.74); DBP: low to high (β = 5.75, 95% CI 3.22, 8.29) and medium to high (β = 3.59, 95% CI 1.64, 5.54)). Furthermore, the association remained when excluding 704 subjects with T2D, obesity, or dyslipidemia at baseline (SBP: low to high (β = 2.27, 95% CI 0.007, 4.53) and medium to high (β = 2.33, 95% CI 0.61, 4.05); DBP: low to medium (β = 3.08, 95% CI 0.31, 5.84), low to high (β = 6.29, 95% CI 3.05, 9.53), and medium to high (β = 3.22, 95% CI 0.75, 5.68)).

### Association between the TyG index and hypertension incidence rate

Of 1,113 participants, 75.9% were females. The characteristics at baseline by TyG index categories were explored. Participants in the top category of the TyG index were older and had higher SBP, DBP, BMI, overweight, and obesity than subjects in the lowest category. In addition, among subjects in the highest category, higher proportions of lipid-lowering and hypoglycemic drugs were observed compared to those in the lowest category (Table S1).

During 10,267 person-years of follow-up, 359 incident cases of hypertension were observed, with an incidence rate of 35.0 per 1000 person-years (95% CI 31.5, 38.8). The crude incidence rate of hypertension in the highest TyG index category was more than twice that in the lowest category (49.4 vs. 22.1, respectively) (Table 4). The Cox proportional hazards analysis showed that the TyG index and hypertension incidence were positively associated. The adjusted model showed that the risk of hypertension in participants in the highest category of the TyG index increased by 56% compared to participants in the lowest category (HR = 1.56, 95% CI 1.18, 2.08). The hazard ratios for hypertension across categories of the TyG index remained similar after adjusting for lipid-lowering drugs (HR = 1.51, 95% CI 1.13, 2.02) and adding hypoglycemic drugs (HR = 1.50, 95% CI 1.12, 2.00) (Table 4). The sensitivity analysis showed that the estimations remained similar. Participants in the top category of the TyG index had a higher risk of hypertension than those in the bottom category when excluding 69 subjects with T2D at baseline (HR = 1.51, 95% CI 1.11, 2.04), 222 subjects with T2D or obesity at baseline (HR = 1.48, 95% CI 1.05, 2.08), and 405 subjects with T2D, obesity or dyslipidemia at baseline (HR = 1.65, 95% CI 1.09, 2.48).

**Table 4:** Risk of hypertension according to categories of the TyG index in participants from HWCS (n = 1,113)

	Triglyceride-glucose index*			
	Low	Medium	High	*P* trend
	n = 372 (33.4)	n = 370 (33.2)	n = 371 (33.3)	
TyG index, median (p25-p75)	8.2 (8.0-8.3)	8.7 (8.6–8.8)	9.3 (9.1–9.6)	
Cases of hypertension	82	124	153	
Person-years	3,709	3,459	3,099	
Crude incidence rate per 1000, (95%CI)	22.1 (17.8, 27.5)	35.8 (30.1, 42.7)	49.4 (42.1, 57.9)	
Model 1, HR (95%CI)	Ref.	1.28 (0.96, 1.69)	1.56 (1.18, 2.08)	0.002
Model 2, HR (95%CI)	Ref.	1.28 (0.96, 1.69)	1.51 (1.13, 2.02)	0.005
Model 3, HR (95%CI)	Ref.	1.28 (0.96, 1.69)	1.50 (1.12, 2.00)	0.006

*Categories defined by tertiles. TyG index: Triglyceride-glucose index

Model 1: Model adjusted for age (years), sex, body mass index (kg/m2), alcohol intake (g/day), physical activity (inactive/active), DASH diet, smoking (never, former, current), family history of hypertension (yes, no, unknown)

Model 2: Model 1 + Lipid-lowering drugs

Model 3: Model 2 + Hypoglycemic drugs

HR: hazard ratio, CI: confidence interval

## Discussion

This prospective study aimed to evaluate the association between the TyG index and SBP, DBP, and hypertension incidence in Mexican adults. Adjusted fixed-effects regression models showed that changing from a low and medium to a high category of the TyG index increases SBP and DBP. This association was also found with DBP when changing from a low to a medium category. Moreover, the adjusted Cox proportional hazards regression models showed that participants in the higher category of the TyG index at baseline had a higher risk of hypertension. Furthermore, the coefficients of both analytic approaches remained similar when excluding T2D, obesity, or dyslipidemia at baseline in the sensitivity analysis. These results suggested that the TyG index predicted the incidence of hypertension. Thus, the early diagnosis of the TyG index could be beneficial for interventions aimed at preventing hypertension among the Mexican population. Consistent with these results, Sánchez et al. found that the TyG index categories defined by quintiles were associated with a higher risk of hypertension (*P* for trend < 0.001) in a Caucasian cohort with an average follow-up of 8.49 years [13]. Moreover, when authors excluded subjects with T2D and obesity at baseline, the HR increased across categories of the TyG index (*P* for trend < 0.001) [13]. In the present study, a similar association when performing the sensitivity analysis excluding T2D, obesity, or dyslipidemia was observed. Another prospective cohort with 9 years of follow-up found that HR increased across categories of the TyG index defined by quartiles (*P* for trend < 0.001) [14]. In addition, six cross-sectional studies conducted with adults suggested a positive association between the TyG index and hypertension [12]. In Mexico, a cross-sectional study conducted with children and adolescents found that an elevated TyG index was associated with hypertension (OR = 1.63, 95% CI: 1.26, 2.11; *P* < 0.001) [26]. For comparison with the previous study, a cross-sectional analysis at baseline (n = 1,545) using a logistic regression model adjusting for confounders was performed, and similar results were found (medium vs. low category: OR = 1.52, 95% CI 1.08, 2.14; *P* = 0.015 and high vs. low category: OR = 1.86, 95% CI 1.32, 2.62; *P* < 0.001). To the best of our knowledge, this is the first study evaluating the longitudinal association between the TyG index and BP in Mexican adults. This study confirms the results of longitudinal population-based studies, which found a positive association between the TyG index and incident hypertension [13, 14].

The TyG index is a substitute to identify insulin resistance [9–11, 27, 28], and it has shown a strong correlation with the hyperinsulinemic-euglycemic clamp test [9], which has been considered the gold standard method for assessing insulin resistance [29]. This index has also shown high sensitivity for recognizing insulin resistance when compared with the homeostasis model assessment (HOMA-IR) index [10]. To evaluate the correlation between the TyG index and HOMA-IR in the present study, Spearman´s correlation was estimated, and the result (*Rho* = 0.591, *P* < 0.001) was higher than that previously reported in another adult Mexican population with similar characteristics (Pearson’s correlation = 0.391, *P* = 0.01) [9]. Furthermore, when Spearman´s correlation in apparently healthy subjects was performed, a higher coefficient (*Rho* = 0.539, *P* < 0.001) than that reported previously in an adult Mexican population with similar characteristics (Pearson’s correlation = 0.322) was found [10]. In addition, a positive association was observed in the present study when evaluating the association with HOMA-IR using a subsample (n = 1,176). When changing from ≤ 3.2 to > 3.2, HOMA-IR categories increased SBP by 2.38 mmHg (95% CI 0.24, 4.52) and DBP by 1.50 (95% CI 0.12, 2.88).

The TyG index has shown an association with metabolic [30, 31] and cardiovascular diseases [32, 33]. Previous findings have suggested the relationship between insulin resistance and hypertension and the possible mechanisms by which insulin resistance may increase BP [5, 34]. First, hyperinsulinemia caused by insulin resistance might increase sympathetic nervous system activity, stimulate adrenaline and norepinephrine secretion, and increase cardiac output and peripheral vascular resistance [35]. The elevated catecholamine might thicken the vascular smooth muscle and induce stenosis and hypertension [36]. Second, insulin resistance may stimulate the renin-angiotensin-aldosterone system (RAAS), produce the reabsorption of H_2_O and Na^+^, and increase vascular activity, eventually leading to hypertension [37, 38]. Furthermore, previous studies have suggested that insulin resistance promotes sodium retention [14, 39]. Additionally, insulin resistance has the potential to enhance the production and release of endothelin, which can lead to the constriction of blood vessels. Moreover, it may reduce the synthesis of prostacyclin (PGI2) and prostaglandin E2 (PGE2), both of which play a role in vessel dilation. Last, insulin resistance could stimulate the growth of vascular smooth muscle cells, contributing to an increase in blood pressure [40, 41]. On the other hand, different studies have shown that elevated levels of plasma triglycerides are associated with a high risk of cardiovascular disease [42]. Hypertriglyceridemia interferes with muscle glucose metabolism and is related to decreasing insulin sensitivity [43, 44]. The product of fasting triglycerides and glucose levels is a substitute measure to estimate insulin resistance; therefore, its usefulness lies in its clinical importance and accessibility [9, 10]. In addition to these mechanisms, there may be other pathways involved in the association between insulin resistance and blood pressure that have not been fully elucidated. Despite adjusting for potential mediators such as obesity, diabetes, and dyslipidemia, the statistically significant associations between the TyG index and SBP/DBP remained, which is consistent with findings from other studies. These findings suggest that there may be additional potential causal pathways that are independent of these factors, and further investigations are warranted to explore these pathways.

### Strength of study

This study had some strengths; the major is that it was a long-term 13-year longitudinal population-based study. This prospective study establishes the temporality between the TyG index and the changes in BP or the development of hypertension in Mexico. Moreover, the fasting triglycerides and glucose were collected directly in each one of the measurements. Repeated measurements also contribute to attenuating potential biases in epidemiological studies.

### Limitations of the study

The present study had some limitations. First, fasting insulin was only obtained in a subset of the samples due to missing reagents, so it was not possible to perform the HOMA-IR analysis with the total sample size. Second, residual confounding from unmeasured or imperfectly measured data could not be ruled out; however, fitted models adjusting for potential confounders were performed. Third, unfortunately, this study had a low proportion of males; therefore, interaction with sex was not explored. Finally, all individuals in this study were enrolled in an urban center; therefore, the conclusions could differ from the general population. Additional studies are necessary to elucidate the above factors due to these limitations.

## Conclusion

The results of this study support the hypothesis that the TyG index is associated with blood pressure in Mexican adults and suggest that interventions aimed at improving triglyceride and glucose levels may positively impact the prevention or control of hypertension. This knowledge may prompt physicians to be more vigilant in blood pressure control and promote lifestyle modifications in patients with higher triglyceride-glucose ratio values. Focus on lifestyle modifications such as diet changes, exercise, and medication management to specifically address triglyceride and glucose control in people at high risk. Understanding the relationship between the triglyceride-glucose ratio and hypertension over a 13-year follow-up period may provide insight into the long-term management of hypertension. This highlights the importance of ongoing monitoring, follow-up, and interventions for maintaining optimal triglyceride and glucose levels in people at high risk of hypertension.

### Electronic supplementary material

Below is the link to the electronic supplementary material.

**Supplementary Material 1: Figure S1**: Flowchart of the study population. **Table S1**: Characteristics of participants in the Health Workers Cohort Study according to the TyG index categories defined by tertiles at baseline (n=1,113)

## Abbreviations

TyG index: Triglyceride-glucose index
BP: Blood pressure
HWCS: Health Workers Cohort Study
IMSS: Mexican Social Security Institute
SBP: Systolic blood pressure
DBP: Diastolic blood pressure
FFQ: Food Frequency Questionnaire
DASH: Dietary Approaches to Stop Hypertension
PA: Physical activity
BMI: Body mass index
T2D: Type 2 diabetes
SD: Standard deviation
HR: Hazard ratio
95% CI: 95% Confidence interval
OR: Odds ratio
β: Regression coefficient
HOMA: Homeostasis model assessment
RAAS: Renin-angiotensin-aldosterone system
