# Supplementary material for: Triglyceride-glucose index is associated with hypertension incidence up to 13 years of follow-up in mexican adults
Source: Lipids Health Dis. 2023 Sep 27;22:162. doi: 10.1186/s12944-023-01925-w (PMC10523648; doi:10.1186/s12944-023-01925-w)
Supplement: Supplementary file 1 — Supplementary Material 1: Figure S1: Flowchart of the study population. Table S1: Characteristics of participants in the Health Workers Cohort Study according to the TyG index categories defined by tertiles at baseline (n=1,113) [file 12944_2023_1925_MOESM1_ESM.docx]

**Table S1.** Characteristics of participants in the Health Workers Cohort Study according to the TyG index categories defined by tertiles at baseline (n=1,113)

| **﻿Population characteristics** | **Triglyceride-glucose index**** | | |  |
| --- | --- | --- | --- | --- |
|  | **Low** < 8.453 | **Medium** 8.454-8.926 | **High** >8.926 | ***P* value ^#^** |
|  | n = 372 (33.4) | n = 370 (33.2) | n = 371 (33.3) | **Low vs high** |
| Age, years * | 40 (31-49) | 45 (37-55) | 47 (41-57) | <0.001 |
| Sex, n (%) |  |  |  |  |
| Females | 315 (84.7) | 294 (78.5) | 236 (63.6) | <0.001 |
| Systolic blood pressure ^†^, mmHg | 109.9 (9.8) | 112.9 (10.1) | 115.6 (9.7) | <0.001 |
| Diastolic blood pressure ^†^, mmHg | 67.7 (8.7) | 69.7 (7.8) | 71.0 (7.7) | <0.001 |
| ﻿Triglycerides *, mg/dL | 85 (70-97) | 130 (115-146) | 217 (183-280) | <0.001 |
| ﻿Fasting plasma glucose *, mg/dL | 85 (80-90) | 90 (84-96) | 95 (89-104) | <0.001 |
| TyG index * | 8.2 (8.0-8.3) | 8.7 (8.6-8.8) | 9.3 (9.1-9.6) | <0.001 |
| Body mass index *, kg/m^2^ | 23.9 (21.7-26.6) | 25.8 (23.5-28.5) | 26.9 (24.7-29.6) | <0.001 |
| Overweight, n (%) | 114 (30.7) | 161 (43.6) | 176 (47.4) | <0.001 |
| Obesity, n (%) | 31 (8.3) | 59 (15.9) | 85 (22.9) | <0.001 |
| Diabetes, n(%) | 5 (1.3) | 16 (4.3) | 48 (12.9) | <0.001 |
| Dyslipidemias, n(%) | 32 (8.6) | 84 (22.7) | 133 (35.9) | <0.001 |
| Smoking, n (%) |  |  |  |  |
| Never | 244 (65.2) | 217 (58.7) | 202 (54.5) | 0.003 |
| Former | 79 (21.2) | 95 (25.7) | 105 (28.3) | 0.024 |
| Current | 49 (13.2) | 58 (15.6) | 64 (17.2) | 0.128 |
| DASH diet ^†^ | 23.6 (4.5) | 23.9 (4.5) | 23.6 (4.4) | 1.000 |
| Alcohol intake *, ﻿g/day | 1.03 (0.21-3.90) | 0.83 (0.21-2.75) | 1.14 (0-21-5.29) | 0.092 |
| Physical activity *, min/day | 14.2 (3.2-40.9) | 12.9 (3.2-36.5) | 12.9 (2.7-33.2) | 0.079 |
| Physical activity ≥30 min/day, n (%) | 138 (37.1) | 128 (34.8) | 119 (32.2) | 0.160 |
| Lipid-lowering drugs, n (%) | 4 (1.1) | 10 (2.7) | 26 (7.0) | <0.001 |
| Hypoglycemic drugs, n (%) | 1 (0.3) | 6 (1.6) | 28 (7.6) | <0.001 |
| ﻿Family history of hypertension |  |  |  |  |
| No | 87 (23.5) | 86 (23.3) | 113 (30.5) | 0.032 |
| Yes | 273 (73.6) | 276 (74.8) | 251 (67.7) | 0.077 |
| Unknown | 11 (2.9) | 7 (1.9) | 7 (1.9) | 0.373 |
| **Categories defined by tertiles * Median (p25-P75) ^†^ Mean (SD) ^#^ For categorical variables, the immediate two-sample proportions test were used and for continuous variables we used Dunn's Test, and for systolic blood pressure, diastolic blood pressure, and DASH diet, one-way ANOVA followed by Bonferroni's post-hoc comparisons tests were performed. TyG index: Triglyceride-glucose index. DASH diet: The Dietary Approaches to Stop Hypertension | | | | |

**Figure S1.** Flowchart of the study population
